# Immune response mechanisms in acute and chronic pancreatitis: strategies for therapeutic intervention

**DOI:** 10.3389/fimmu.2023.1279539

**Published:** 2023-10-10

**Authors:** Juliane Glaubitz, Saeedeh Asgarbeik, Rabea Lange, Hala Mazloum, Hager Elsheikh, Frank Ulrich Weiss, Matthias Sendler

**Affiliations:** Department of Medicine A, University Medicine, University of Greifswald, Greifswald, Germany

**Keywords:** acute pancreatitis, chronic pancreatitis, immune response, fibrosis, therapy

## Abstract

Acute pancreatitis (AP) is one of the most common inflammatory diseases of the gastrointestinal tract and a steady rising diagnosis for inpatient hospitalization. About one in four patients, who experience an episode of AP, will develop chronic pancreatitis (CP) over time. While the initiating causes of pancreatitis can be complex, they consistently elicit an immune response that significantly determines the severity and course of the disease. Overall, AP is associated with a significant mortality rate of 1-5%, which is caused by either an excessive pro-inflammation, or a strong compensatory inhibition of bacterial defense mechanisms which lead to a severe necrotizing form of pancreatitis. At the time-point of hospitalization the already initiated immune response is the only promising common therapeutic target to treat or prevent a severe disease course. However, the complexity of the immune response requires fine-balanced therapeutic intervention which in addition is limited by the fact that a significant proportion of patients is in danger of development or progress to recurrent and chronic disease. Based on the recent literature we survey the disease-relevant immune mechanisms and evaluate appropriate and promising therapeutic targets for the treatment of acute and chronic pancreatitis.

## 1 Introduction

Acute pancreatitis is an inflammatory disease of the pancreas which constitutes, with an annual incidence rate of ~34 per 100,000 people, one of the most common diseases of the gastrointestinal tract (1). A significant proportion of patients which experience an episode of acute pancreatitis (about 20-30%) develop a chronic form of the disease (2). Chronic pancreatitis (CP) is defined as a fibroinflammatory disorder of the pancreas, which is mostly characterized by recurrent episodes of AP, and results in a progressive loss of exocrine and endocrine tissue (3). Acute and chronic pancreatitis are clinically defined pictures of a common underlying disease, where immune response mechanisms orchestrate the repair, resolution or fibrotic replacement of acinar cell damage. Acute pancreatitis is defined by a self-digestion of pancreatic tissue by its own proteases (4). The most important etiologic factors include gallstones, alcohol abuse, genetic risk, trauma, metabolic disorders, drugs, infections, and autoimmune causes. The intra-acinar activation of trypsinogen to trypsin has been hypothesized as the initial triggering event in the disease process (5),which ultimately leads to necrotic cell death of acinar cells (6). In recent years, however, it has become increasingly clear that also trypsin-independent cellular mechanisms can initiate cell damage and are thus causative for the development of pancreatitis (6). Genetic functional analyses of sequence variants identified in patients with chronic pancreatitis (CP) unravelled the cellular pathomechanisms which are involved in acinar cell damage. In addition to mutations related to trypsin activity (7–9), misfolding mutations were detected that cause increased endoplasmic reticulum (ER) stress (10–12). Mutations in the cystic fibrosis transmembrane conductance regulator (CFTR) are also related with an increased CP risk and were found to associate with decreased pancreatic secretion (13–15). Whereas these roles of genetic risk factors are relatively well studied, the cellular pathomechanism behind ethanol-toxic pancreatitis is largely not understood. Another major cause is an obstruction of the pancreatic duct by bile stones, but the cellular mechanisms here have also not been fully elucidated. It is assumed that an increased pressure build-up in the pancreatic duct triggers the opening of pressure-sensitive Ca^2+^ channels and results in cytoplasmatic Ca^2+^ overload leading to intracellular protease activation (16).

Independent from etiology and triggering factors of an acute episode of pancreatitis we observe an immediate response of the immune system to local cell damage which ultimately defines the further course of the disease. Acinar cells activate the transcription factor NFκB in parallel to the Ca^2+^ signal and initiate the expression of cytokines and chemokines which start the local immune response. They mobilize cells of the innate immune system such as monocytes and neutrophils from the bone marrow, and recruit them into the damaged tissue area of the pancreas (17). Tissue resident macrophages are among the first cells of the innate immune system which recognize damage associated molecular pattern (DAMPs) and cytokines released from acinar cells and enhance the initial local immune response. After the onset of the local response more immune cells start to migrate into the organ where they get activated by DAMPs (18, 19). Activated leukocytes in turn stimulate the pro-inflammatory response as well as the local damage by releasing further cytokines, such as TNF-α (20) or reactive oxygen species (ROS) (21). In the later stage of the disease, the inflammatory response changes from a pro-inflammatory toward an anti-inflammatory phenotype which is a prerequisite for wound healing and fibrogenesis (22, 23). Repeated acute episodes frequently progress to chronic pancreatitis which associates with a continuous fibrotic replacement of exocrine and endocrine tissue.

Whereas triggering causes of pancreatitis can vary, the immune response to cell damage is identical and therefore may be best suited for clinical intervention. Due to the complex and dynamic regulation of the immune response in the disease course, any intervention needs to be well balanced and done with caution. In the following we will review in more detail the involvement and therapeutic potential of important signaling pathways and regulation mechanisms in acute and chronic pancreatitis. About 20% of acute pancreatitis patients develop a severe disease course with systemic complications, peripheral organ failure or the infection of pancreatic necrosis by commensal intestinal bacteria (24). Severe disease courses are caused by derailed or unbalanced immune responses and are associated with high mortality and morbidity. The treatment of severe acute pancreatitis is a clinical challenge because the therapy needs to target complex immune regulation mechanisms, which mostly have been investigated in animal models. The translation of therapeutic strategies into the clinic is promising, but also needs to consider existing differences of mouse and human immunity.

## 2 Acute pancreatitis

### 2.1 NFκB initiates the inflammatory response

The onset of acute pancreatitis is characterized by an induction of cell damage in the exocrine pancreas, which ultimately results in acinar cell death. Caused by intracellular Ca^2+^ signaling and the cell damage, the transcription factor NFκB becomes activated (17). NF-κB is found in almost all cell types and plays a key role in regulating inflammatory responses to stimuli such as stress, cytokines, free radicals, ultraviolet irradiation, danger associated molecular patterns and bacterial or viral antigens. NF-κB controls inflammation not only directly by increasing the production of inflammatory cytokines, chemokines and adhesion molecules, but also by regulating the cell proliferation, apoptosis, morphogenesis and differentiation. Therefore, NFκB could be a possible therapeutic target to control the onset of the immune response and to mitigate the disease progression at an early stage. There are several clinically well-studied and approved inhibitors of the NFκB pathway available, such as aspirin or cyclosporine A (25). Experimental work has shown that NFκB is involved not only in the regulation of immune-related genes, but also in an anti-stress response (26). In an experimental pancreatitis model in mice, the acinar cell specific knockout of *RelA*, coding for the p65 subunit of NFκB, significantly increased disease severity (27). Likewise, the acinar cell-specific knockout of IκBα, an intracellular inhibitor of NFκB phosphorylation, was shown to increase NFκB activity and resulted in a milder disease course (26). The effect is different when we look at NFκB activation in leukocytes. In pancreatic macrophages, or bone marrow-derived macrophages (BMDMs), NFκB activation stimulates the release of cytokines and chemokines (18), which drives the systemic hyperinflammation and generates a “cytokine storm”. Apparently, signaling pathways can have cell specific effects, which limits their eligibility for a broad pharmacological intervention. Instead, both, the molecular target, as well as the target cell must be carefully selected and specifically addressed analogous to a targeted cell-specific tumor therapy.

### 2.2 The local immune response

**Figure 1:**
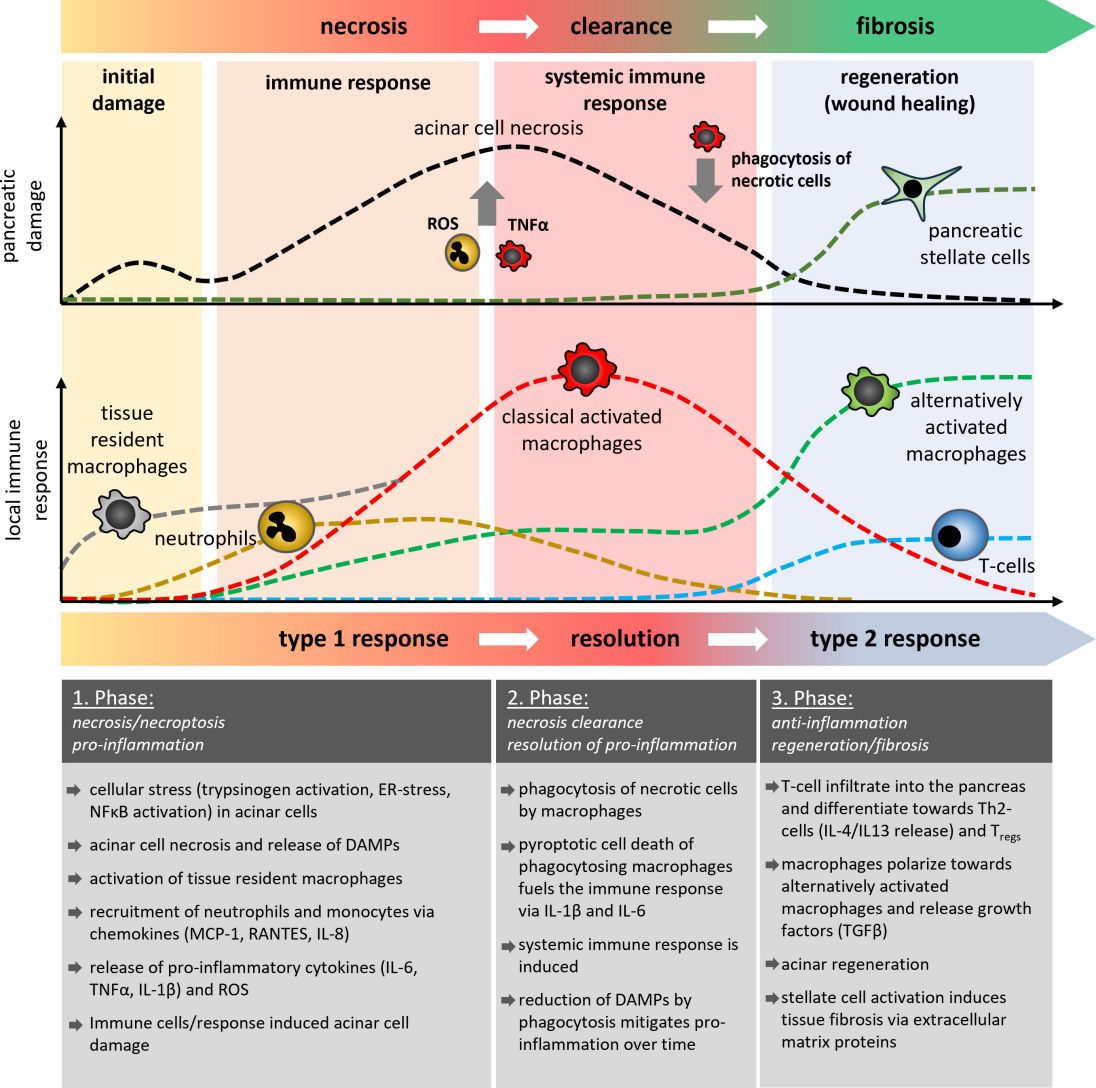
Schematic illustration of the temporal development of pancreatic damage and the local immune response during acute pancreatitis. In three different phases of the disease course, it is shown how cells of the innate and adaptive immune system are involved in the processes of acinar necrosis, the clearance of damaged tissue and the regeneration of pancreatic tissue or it`s fibrotic replacement. Tissue resident macrophages (grey) are the first cells which become activated and differentiate into classical or alternatively activated macrophages, similar to the monocyte derived macrophages. The AP-induced immune response showed similarities to classical wound healing reactions which results in fibrosis and scar formation. Multiple episodes of AP thus result in increased organ fibrosis which characterises the clinical picture of CP.

Based on observations from the mouse model of caerulein-induced pancreatitis, the local damage scenario in the pancreas has two temporally separated peaks (4, 5, 28). The first peak comprises the acinar cell damage (5, 29), which is caused by intracellular protease activation or massive ER stress and occurs within the first hour after induction of the disease. The second peak of damage is mainly triggered by infiltrating leukocytes (18, 20–22, 30) and is significantly more severe as it can develop into systemic complications (**Figure 1**). Animal studies have shown that cells of the innate immune response, such as neutrophils and macrophages rapidly migrate into the pancreas after the onset of the disease (20, 21). In contrast, the cells of the adaptive immune system, such as CD4^+^ and CD8^+^ T-cells migrate significantly later into the inflamed pancreas (31, 32). They have a crucial role in the fibroinflammatory process during chronic pancreatitis (33–35). In the first step damaged acinar cells initiate an immune response via NFκB activation and the release of cytokines and chemokines which recruit leukocytes to the side of inflammation. Chemokines such as MCP-1, RANTES, MIG-1, IP-10, and IL8 are significantly elevated in the serum of patients and attract cells to the pancreas (36–38). In parallel to the chemokine mediated cell recruitment, necrotic acinar cells trigger via DAMPs the activation of tissue resident macrophages and initiate the release of cytokines (39). Tissue resident macrophages represent a significant population of quiescent immune cells in the exocrine and endocrine pancreas which react immediately on appearing damage signals (39). These activated local macrophages start a self-enhancing immune response by secreting pro-inflammatory cytokines and chemokines to recruit more immune cells to the side of inflammation (18, 22, 40). However, it is important to note that tissue resident macrophages are also essential to induce cell regeneration and therefore are necessary to minimize the acute damage during pancreatitis. Recent work has shown that especially the induction of fibrosis is a critical protective factor for the survival of severe acute pancreatitis. Depletion of tissue resident macrophages by CSF1 Antibody + clodronate containing liposomes significantly reduced the survival of animals and impaired the tissue regeneration after acute pancreatitis (41). It seems that macrophages have opposing roles in pancreatitis, therefore, a therapeutic treatment involving macrophages must be applied with caution as it could be a double-edged sword.

Neutrophils are among the first immune cells which are recruited to the damaged organ. In contrast to macrophages, they are not tissue resident cells, but extravasate from the vascular system. Antibody-mediated depletion of neutrophils in animal models of acute pancreatitis significantly ameliorated the disease course (20, 21, 38). A crucial role in this process plays the excessive release of reactive oxygen species (ROS) via oxidative burst, a defense mechanism against pathogenic bacteria (42). The excessive release of ROS causes additional damage of pancreatic tissue (21) and also damages the vascular system, which in turn stimulates the migration of more immune cells (42). Another defense mechanism of neutrophils against pathogens is the formation of net-like chromatin fibers decorated with antimicrobial proteins, which trap and kill bacteria to prevent further spreading in the organism (43). NETs (Neutrophile extracellular traps) are also formed during acute pancreatitis, and animal studies have shown that these NETs increase the local damage and therefore could be a potential therapeutic target (44, 45). Leppkes et al. analyzed the underlying pathomechanism and showed that NETs are involved in stone formation and contribute to pancreatic duct obstruction (46). They proclaim that one of the main triggers for NET formation is the pro-inflammatory cytokine IL-17A, which is released by a subset of pro-inflammatory T helper cells (Th17 cells). T-cell infiltration into the pancreas occurs in the middle or later phase of the disease, but the activation of T-cells can be observed in the lymphoid tissue (40) or the small intestine (47, 48), where they play a crucial role for the intestinal barrier function. Beside IL-17A also pathogens, PAMPs and DAMPs can induce NET formation in neutrophils (49)however, AP is a primarily sterile inflammation, so it seems unlikely that pathogens or PAMPs cause NET formation in AP Mechanical stimuli such as microcrystals can also trigger netosis and have been found to play a crucial role in the formation of gallstones from biliary crystals (50). They could also be a direct cause of AP, independent of Th17-cells. IL-17A acts directly on neutrophils and recruits them to the side of inflammation (51). A limited T-cell infiltration in the early phase of the disease may provoke also a limited neutrophil migration (18). Neutrophils are rather out of place during pancreatitis, because in the beginning, pancreatitis is a primarily sterile inflammation and neutrophils are mainly involved in bacterial clearance. Still, their main function is to trap and kill bacteria via NETs and ROS release, and both functions increase the local organ damage during pancreatitis (21, 46) (**Figure 1**).

In contrast to neutrophils, monocytes react more flexible to the local circumstances. Chemokines like CCL2 (MCP-1) are involved in the mobilization and recruitment of monocytes. CCL2-binding to its receptor CCR2 recruits mainly pro-inflammatory Ly6C^hi^ monocytes, but also neutrophils and T-cells to the site of inflammation (52). While circulating monocytes migrate to the pancreas, tissue-resident macrophages are the first immune cells that react immediately to the pancreatic damage. A therapeutic blockade of the CCR2 pathway diminishes the migration of monocytes and reduces the local as well as the systemic damage (38, 53, 54). Notably, this blockade does not significantly affect the local macrophage response, as tissue resident macrophages start to proliferate and are able to substitute the monocyte derived macrophage response (38). Monocyte derived macrophages enter the pancreas almost as fast as neutrophils (18, 20, 38), but in contrast to neutrophils, macrophages are, in addition to a bacterial clearance also responsible for the removal of cellular debris and dead cells. In acute pancreatitis macrophages phagocytose the remains of damaged or dead acinar cells (18, 55). Crucial for the clearance process is the nature of the damage (56, 57). Apoptotic cell bodies are immunologic silent, as they do not induce a pro-inflammatory response (57). Necrotic cells are different, because they release intracellular organelles and proteins which act as DAMPs and induce a pro-inflammatory phenotype of macrophages similar to the response to pathogen associated molecular pattern (PAMPs) (56). In pancreatitis, a switch from necrotic cell death to apoptotic cell death can ameliorate the disease course (58), but acinar cell death is mainly due to necroptotic cell death (59). Necroptosis is a form of regulated necrosis, which is induced by TNF-α via the TNF receptor signaling pathway (60). Like in necrosis it leads to the release of DAMPs, which induce the pro-inflammatory macrophage phenotype and cause secretion of cytokines TNF-α, TRAIL, IL-6, IL-1β, IL-18 and chemokines such as CCL2, CCL5, CXCL3 and IP-10 (18, 22, 40). Consequently, this initiates a self-sustaining cycle of TNF-α-secreting macrophages which in turn induce necroptosis of acinar cells and the release of more DAMPs to trigger the proinflammatory reaction. Pro-inflammatory macrophages secrete the majority of cytokines which fuel the upcoming cytokine storm and cause the manifestation of the inflammation (6, 18, 20, 22, 30, 40). The extent of the initial acinar cell damage seems critical in shaping the macrophage response, which in turn enhances the local damage and triggers the development of a cytokine storm and systemic complications. As macrophages are essential for the clearance of the damaged cells in the organ, they cannot be easily targeted in a therapeutic approach (55). Instead, the released cytokines, which play a major role in controlling the systemic immune response, could be addressed (**Figure 1**).

### 2.3 Cytokine storm

TNF-α looks like a promising target to interrupt this vicious cycle, depletion of TNF-α reduced the pancreatic damage via inhibiting necroptosis and diminished the pro-inflammatory response (60). A depleting antibody (Infliximab) is already in clinical use for the treatment of rheumatoid arthritis (61), and an ongoing study currently investigates the therapeutic potential of infliximab in patients with acute pancreatitis (NCT03684278). Previous studies on patients suffering from sepsis or Morbus Crohn showed an increased risk of secondary infections, and the mortality risk was significantly higher in sepsis patients (62, 63). An extended blockage of pro-inflammatory signaling could strengthen the anti-inflammatory response and increase the risk of secondary infections, which are known to be a serious risk also during severe acute pancreatitis (24).The current study will show if anti-TNF-α treatment is effective and safe in pancreatitis patients.

In addition to TNF-α, activated macrophages secrete also IL-6, which is another well-known pro-inflammatory cytokine (64). In acute pancreatitis, increased serum levels of IL-6 correlate with disease severity (40, 65). In addition to an activation of the classical IL-6 signaling pathway through the IL-6 receptor/gp130 complex, IL-6 can also act via the IL-6 trans-signaling pathway by interaction with the soluble IL-6 receptor on cells that do not express the classical IL-6 receptor, but only gp-130 (66).. Trans-signaling can have dramatic effects on various cell types. During pancreatitis, IL-6 trans-signaling causes lung damage (67) and defines the systemic disease severity. ADAM17 cleaves the membrane bound IL-6R to its soluble form sIL-6R, which is essential for the IL-6 trans signaling and enables the binding to the gp130-receptor pathway in the absence of membranous IL-6R. The inhibition of ADAM17 results in decreased IL-6 trans signaling and was shown to ameliorate the disease severity of acute pancreatitis (68). Tocilizumab, an antibody which blocks the classical IL-6 pathway by binding to the IL-6 receptor is approved for the treatment of rheumatoid arthritis (69) and was also tested in an animal model for the treatment of acute pancreatitis with promising results (70).

Another proinflammatory cytokine released by macrophages in the course of acute pancreatitis is Interleukin 1β (71). Pancreas-specific overexpression of IL-1β results in a form of chronic pancreatitis and causes a complete disintegration of the organ (72). IL-1β is mainly released by classically activated macrophages. The secretion of IL-1β is rather complex and requires activation by two independent signals: 1) the induced expression of inflammasome components and of pro-IL-1β via the transcription factor NFκB, and 2) the initiation of the inflammasome complex formation, consisting of NLRP3, ASC and caspase 1. Only this inflammasome complex is capable of processing pro-IL-1β by caspase 1 to its mature form. After phagocytosis of necrotic cell debris (18), macrophages express and activate a cytosolic inflammasome complex, which starts the proteolytic maturation of pro-IL-1β and pro-IL-18 (18, 19, 40). Both cytokines are released from the cytoplasm into the extracellular space by the formation of the gasdermin-D pore complex. Cleavage of gasdermin D by caspase 1 is an essential prerequisite in the pore formation and leads to pyroptotic cell death of the macrophage. Recent data have shown that IL-1β maturation by the inflammasome complex is a key driver of pro-inflammation, pancreatic tissue damage (71) and systemic inflammation (40, 73). Inflammasome related pathways are accessible by multiple treatment options. The NLRP3 inhibitor MCC950 inhibits the inflammasome complex formation (74), and was shown to ameliorate the disease severity in an animal model of acute pancreatitis (40). Also, treatment with lactate (73, 75) or butyrate (76), which are both known to supress inflammasome activation, are similar effective. The inhibition of the IL-1β signaling pathway is another treatment option (77). The IL1 receptor antagonist Anakinra, the soluble decoy receptor rilonacept and the neutralizing antibody canakinumab have been tested in multiple diseases like recurrent pericarditis (78) and rheumatoid arthritis (79) or autoinflammatory diseases such as the familial Mediterranean fever (80). Beside these chronic diseases, the IL1 receptor antagonist Anakinra showed beneficial effects also in severe acute conditions like in sepsis patients with features of the macrophage activation syndrome (81). However, not all patients benefited from the treatment, especially when the immunosuppression was too intense (82). The immune mechanisms of severe acute pancreatitis and sepsis are similar, so the therapeutic treatment of the IL1 pathway could be promising, but no clinical studies with AP patients have been performed to date. One single study in a rat model of acute pancreatitis showed that the application of anakinra had a beneficial effect on disease severity (83). Clinical trials are necessary to further evaluate the therapeutical potential of these drugs.

Besides pro-inflammatory cytokines also the anti-inflammatory cytokine Interleukin 10 is increased in serum of patients with AP (65, 84). IL-10 has immunosuppressive functions and inhibits the secretion of pro-inflammatory cytokines via the induction of SOCS3, which would make a stabilized IL-10 protein derivate a good candidate for a clinical treatment (85). IL-10 can reduce the severity of acute pancreatitis in animal models (86, 87), furthermore, it has been shown that the preventive administration of IL-10 can reduce the risk of post-ERCP pancreatitis (88). Notably, IL-10 treatment also comes with a risk; while it can help to control the SIRS, excessive suppression of the immune response can lead to CARS or even immune paralysis. In the course of severe AP we observe a particular and excessive activation of regulatory T cells, which act immunosuppressive via the release of IL-10 (40, 65) and are responsible for the translocation of commensal bacteria from the intestine (47); additional administration of IL-10 could enhance this effect. The induction of a hypo-inflammation can facilitate the translocation of enteral bacteria to pancreatic necrosis and is associated with high mortality, as infected necrosis is one of the highest risk factors for severe disease and fatal outcome of acute pancreatitis (24).

### 2.4 SIRS/CARS – balance

**Figure 2:**
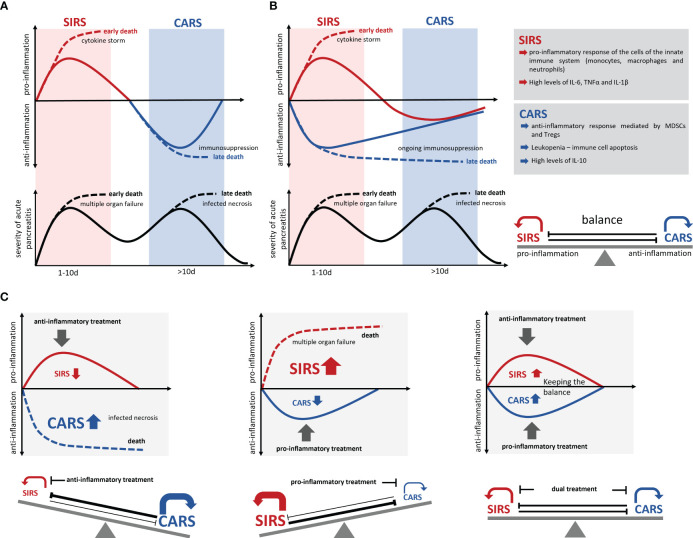
Development of SIRS/CARS during acute pancreatitis. **(A)** Illustrates the temporal correlation of SIRS and CARS with the disease progression. While SIRS frequently leads to organ complications and associates with early mortality, infected necrosis frequently occurs during CARS and causes late mortality. Excessive pro-inflammation suppresses the anti-inflammation and results in an immunological disbalance which is associated with serious complications and an increased mortality (dotted lines). However, excessive anti-inflammation also results in a suppressed ability to respond to bacterial infection and leads to colonization of necrosis by commensal intestinal bacteria, which is also associated with a severe disease course and increased mortality (dotted lines). **(B)** Recent data suggest a simultaneous induction of pro- and anti-inflammatory responses, which keep the balance during the disease progression. **(C)** An immunotherapeutic approach needs to preserve this balanced immune response and prevent a shift to either SIRS or CARS. Solid lines mark the immune dysbalance and disease severity of surviving patients, whereas dotted lines indicate a lethal course of the disease.

Development of a cytokine storm, meaning the uncontrolled and excessive release of pro-inflammatory cytokines, can cause serious complications like multisystem organ failure and death (89). Parallel to the massive release of pro-inflammatory cytokines the levels of anti-inflammatory cytokines like IL-10 also rise in a severity dependent manner (40, 65) as it is observed in sepsis (90). The reason behind is an insidious immunological counter-regulation, the so called compensatory anti-inflammatory response syndrome (CARS), a systemic deactivation of the immune system tasked with restoring homeostasis from an inflammatory state. In the past, it was assumed that CARS builds up as a consequence of the excessive pro-inflammation of SIRS. The increasing auto-immunosuppression in CARS in the worst case, can lead to immune paralysis, which is also associated with serious complications such as infection of pancreatic necrosis by commensal intestinal bacteria (**Figure 2A**). Pro-inflammatory mediators such as IL-6, IL-12 or IL-1b which are released during the cytokine storm suppress an anti-inflammatory response (40, 91, 92), and thus further the pro-inflammation. On the other hand, anti-inflammatory mediators such as IL-10 suppress the pro-inflammatory response and enhance the type 2 immune response via the expansion of Tregs, M2 macrophages and Th2 cells (40, 47, 93). Immune response mechanism need to keep a balance between pro- and anti-inflammatory mediators to prevent life-threatening complications during severe AP (**Figure 2**). Recent studies on sepsis or severe trauma from burn injuries suggest that CARS already develops in parallel with SIRS (91, 92, 94, 95). Matching with the cytokine storm which originates from the local macrophages (18, 19, 22), a systemic counter-regulation is observed (40, 65) (**Figure 2B**). Early in the disease course an increase of regulatory T-cells (47), Th2 cells (40) and myeloid derived suppressor cells (MDSCs) (65) can be seen and IL-10 levels start to rise (40, 65). Whereas SIRS has clear diagnostic criteria (96), CARS is difficult to evaluate in patients. Lymphocyte dysfunction or increased apoptosis cannot be directly measured for the clinical diagnosis (97), so they often occur unnoticed. Further, increased serum levels of IL-10 are found in patients with severe traumata (92, 98) as well as in patients with severe acute pancreatitis (40, 65). Experimental data show that IL-10 can be protective and is able to reduce the severity of pancreatitis by limiting pro-inflammation (86). A critical limit, where pro-inflammation switches to immunosuppression is hard to define. The fact that IL-10 increases in parallel with pro-inflammation and that there are no reliable diagnostic marker to detect hypo-inflammation at an early stage makes the prediction of severe disease courses very difficult and unreliable. Leukopenia and decreasing pro-inflammation are clear signs of an ongoing CARS, but may, at the timepoint of detection, be too advanced to prevent immune paralysis and the development of secondary infections (24). Improved early diagnosis of CARS is essential for an effective treatment.

The fact that pro-inflammatory and anti-inflammatory mechanisms are active at the same time, suggests that the balancing of the immune response is crucial for the outcome (91, 94, 99). Most therapeutic agents are directed against the excessive pro-inflammation in order to mitigate SIRS and its associated complications. These approaches are unfortunately also effective in increasing the counter-regulation (CARS) and thus promoted secondary infections. Similarly, anti-inflammatory therapies would increase the pro-inflammatory response and further advance the SIRS (90). A critical factor in the counter regulation of SIRS is a stringent time control, meaning rapid start and rapid cessation of the treatment. Due to the long-lived nature of neutralizing antibodies such as infliximab (anti-TNF-α), tocilizumab (anti-IL-6 receptor), or canakinumab (anti-IL-1β), the treatment cannot be terminated at once. In contrast, small molecule inhibitors such as MCC950 (NLRP3 inhibitor) have a much shorter half-life and are rapidly excreted after termination of the therapeutic application.

A balanced treatment of both, pro- and anti-inflammatory responses is probably the most promising therapy option for AP (**Figure 2C**). The choice of therapeutic targets is crucial to maintain the balance between hyperinflammation and hypoinflammation, as various cytokines directly affect SIRS as well as CARS. One example of this two-sided activity is TNF-α which is instrumental in the establishment of pro-inflammation, but on the other side indirectly and in combination with FasL and TRAIL triggers apoptosis via the TNF receptor signaling pathway (100, 101). A similar phenomenon is observed with IL-1β, which is decisively involved in the pro-inflammatory reaction, but simultaneously induces T-cell apoptosis in the intestinal lamina propria, and promotes secondary infections (102). During severe acute pancreatitis the activation of immunosuppressive regulatory T-cells causes a decrease in the number of systemic T-effector cells (31, 40, 47). The experimental depletion of regulatory T-cells in an AP mouse model resulted in a stabilization of the T-effector cell population and prevented infection of necrosis (47). To focus on one specific therapeutic target may be insufficient to rebalance the immune response, therefore parallel approaches should be considered.

Infection of pancreatic necrosis by commensal intestinal bacteria is the most serious complication of acute pancreatitis (24). In the infected necrotic tissue 16S rRNA sequencing detected the presence of facultative pathogenic bacteria, which migrated from the duodenum into the pancreas (47). Recent data show that pathogenic bacteria multiply during acute and chronic pancreatitis (47, 103). It is known that the composition of the intestinal microbiome has a significant influence on the immune response which may implicate a certain therapeutic potential of probiotic food or supplements (104). Animal data have demonstrated that antibiotic iradication of the gut microbiome delays pancreas carcinogenesis by shifting the T-cell response in the tumor (105, 106). The microbiome/inflammation axis could also influence the disease course of acute and chronic pancreatitis (47, 107, 108). In this context the therapeutic administration of antibiotics needs to be re-evaluated and reconsidered.

## 3 Chronic pancreatitis

Recurrent episodes of pancreatitis, cause morphological changes and the development of functional deficits which lead to the diagnosis of chronic pancreatitis. The clinical pictures of recurrent acute episodes and chronic pancreatitis are very similar, and a relevant percentage of recurrent acute pancreatitis patients develop chronic disease over time. This suggests a general and progressive underlying disease mechanism that needs to be targeted by therapeutic intervention. Chronic pancreatitis is frequently characterized by recurrent episodes of acute inflammation increasing over time the fibrotic replacement of exocrine and endocrine cells (3). In every acute episode the development of a severe course has to be prevented and the intra pancreatic immune homeostasis must be balanced between a pro-inflammatory response and the regenerative and pro-fibrotic reaction.

**Figure 3:**
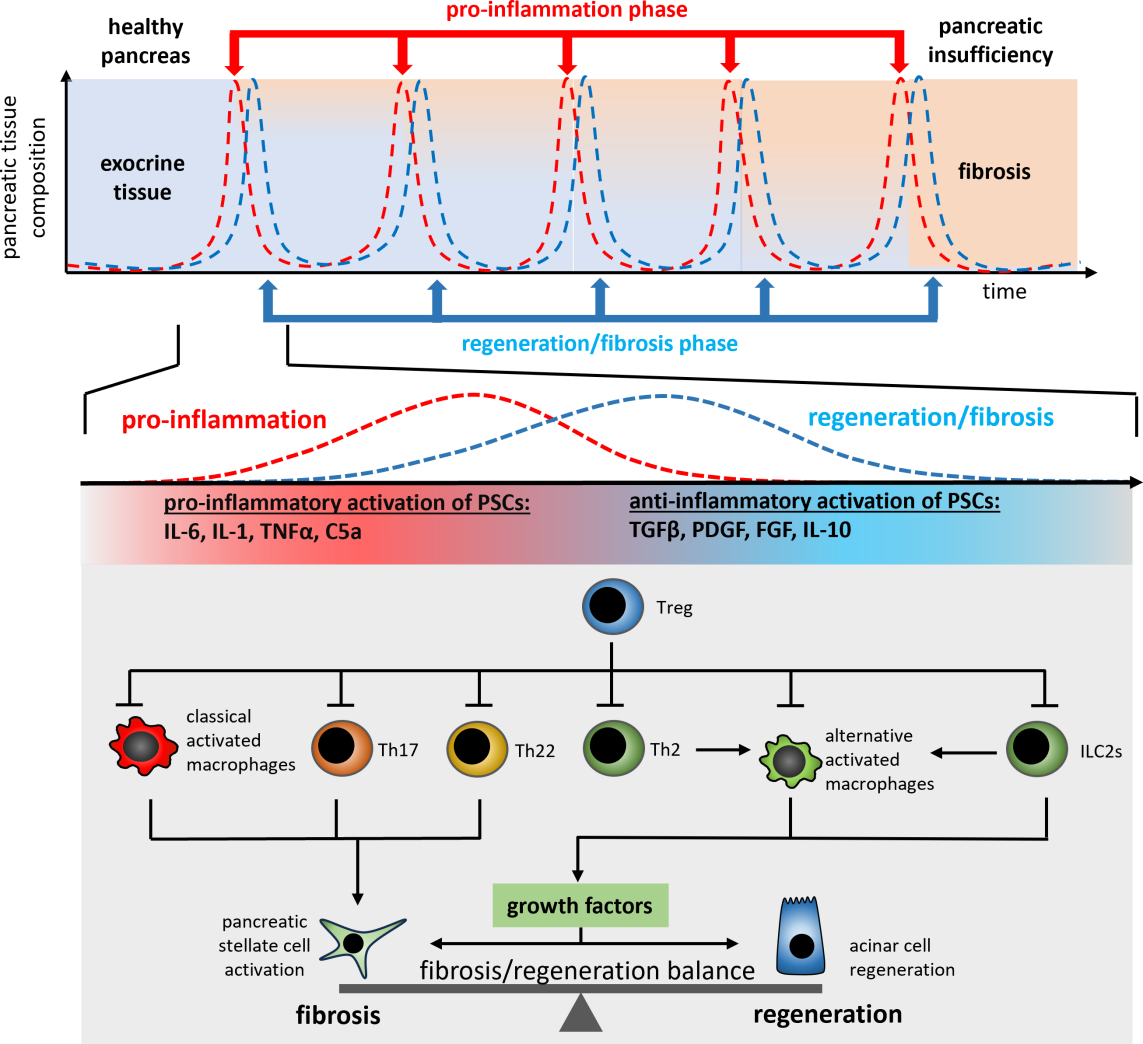
A schematic illustration of the regulation mechanism of cell regeneration vs. fibrogenesis in recurrent acute episodes of chronic pancreatitis patients. Each repetitive episode of pro-inflammation and wound healing or fibrosis is characterized by the release of PSC activating pro-inflammatory cytokines such as TNFα or IL-1β from macrophages and T-cells (Th17, Th22). The anti-inflammatory type 2 immune response (Th2 and ILC2s) in parallel induces the differentiation of alternatively activated macrophages which control the fibrosis/regeneration balance via the release of growth factors like TGF-β and PDGF. Both, pro- and anti-inflammatory signals are involved in the control of the regeneration/fibrosis balance.

Progredient fibrosis of the pancreas goes along with the loss of exocrine and endocrine tissue. Over time, more and more acinar cells are destroyed in the acute inflammation phases, and are replaced by fibrotic tissue (**Figure 3**). The type 2 immune response which is activated in the acute phase in general is responsible for tissue regeneration and wound healing (40). From this point of view the destruction of acinar cells causes a severe, but primarily sterile wound in the pancreatic tissue, which is characterized by a high degree of necrosis and necroptosis (59). As in the case of external wounds, the wound closure is initiated immediately to prevent infection and here the question arises if the fibrotic replacement should be prevented by a specific treatment? On the one hand, fibrosis is part of the regeneration process leading to wound closure. Recent data has shown that fibrosis induced by tissue resident macrophages is essential for the survival of the acute episode of pancreatitis and that fibrosis also plays a protective and regenerative role (41). On the other hand, excessive fibrosis over time can lead to the displacement of exocrine and endocrine tissue and may result in pancreatic insufficiency.

### 3.1 The pancreatic infiltrate

After necrotic tissue areas in the pancreas have been cleared by phagocytosing macrophages the absence of DAMPs interrupts the inflammatory cascade and the classical activation of macrophages changes to an alternatively activated phenotype (22, 23, 35). Due to the absence of cytokine and chemokine release neutrophil granulocytes are no longer recruited to the pancreas and the resident neutrophils have only a short half-life time and vanish from the organ. Cells of the adaptive immune system are now detectable in high numbers and a distinct population of resident CD4^+^ and CD8^+^ T-cells can be observed (33–35). Beside the infiltration of T-cells also innate lymphoid cells type 2 (ILC2) populate the fibroinflammatory infiltrate (35). The presence and activation status of these immune cells ultimately results in the activation of pancreatic stellate cells and fibroblasts, which drive pancreatic fibrosis and the production of extracellular matrix proteins.

### 3.2 Alternative activated macrophages activate pancreatic stellate cells

In response to changing microenvironment (LPS, IFNy or cytokines) macrophages can change their phenotype to fulfil different functions. Activated macrophages are usually divided into two categories, M1-like macrophages and M2-like macrophages, with M1 macrophages being mainly involved in pro-inflammatory responses and M2 macrophages (alternatively activated macrophages) being involved in anti-inflammatory responses. Alternatively activated macrophages are the most intensely studied population of immune cells during chronic pancreatitis. M2 macrophages can be further subdivided into different subclasses (M2a, M2b, M2c and M2d) based on the expression patterns of cell surface markers and their release of pro- or anti-inflammatory cytokines (109). During chronic pancreatitis, M2a macrophages, which are characterized by the expression of CD206, ARG1 and FIZZ1, support cell regeneration and fibrosis by releasing TGFβ and IL-10. M2b macrophages, on the other side, are activated via TLR signaling or IL-1β and mainly support the Th2 response by IL-10 secretion Also, M2c macrophages act immunosuppressive and are responsible for the phagocytosis of apoptotic cells, whereas M2d macrophages release IL-10 and induce vascularization via secretion of VEGF. Alternatively activated macrophages (M2) play an important role in the progressive fibrosis of the pancreas (23) but are also involved in organ regeneration (110). The polarization of alternatively activated macrophages (M2a) is primarily induced by the Th2 cytokines IL-4/IL-13, which cause phosphorylation and activation of the transcription factor STAT6 via the IL-4/IL-13 receptor. These alternatively activated macrophages secrete various growth factors including TGF-β, and activate pancreatic stellate cells (PSC) as well as acinar cell regeneration (111). Stellate cells are mainly responsible for fibrogenesis during chronic pancreatitis. Following activation by various growth factors like TGF-β and PDGF (112) or cytokines such as IL-6 and TNF-α (113) they secrete extracellular matrix proteins (114). Like leukocytes, stellate cells migrate from the bone marrow into the damaged pancreas (115, 116). Ino et al. were able to show that monocytes, which migrate into the pancreas via the MCP-1/CCR2 signaling pathway, can differentiate into PSCs (117). In turn, PSCs also influence the immune response and secrete cytokines and chemokines such as MCP-1 to further recruit monocytes into the organ (118). Likewise, they can also influence the polarization of macrophages to an alternatively activated phenotype via the secretion of IL-4/IL-13 (23). This immune cell-like behavior shows their critical involvement in the immune response during chronic pancreatitis and the direct connection of tissue fibrosis with the immune response. The IL-4/IL-13 axis is the most intensively studied signaling pathway affecting pancreatic fibrosis. Therapeutic blockade of the IL-4 receptor (23), or even the use of a depleting IL-4 antibody (35), results in significantly reduced fibrosis of the pancreas. Pascolizumab, an IL-4 depleting antibody is admitted for clinical intervention in moderate to severe asthma (119). Dupilumab, an IL-4/IL-13 receptor blocking antibody, is used in dermatitis as well as asthma (120, 121). Neither of these agents have so far been clinically tested in chronic pancreatitis. One reason for this being the immunosuppressive effect of the therapy, which could increase the risk of developing infected necrosis during an acute episode of pancreatitis. Furthermore, acute pancreatitis was reported as a very rare side effect of treatment with Dupilumab (122).

Macrophage polarization is mainly regulated by T-cells, ILCs, and PSCs. Folias et al. described a significantly reduced number of alternatively activated macrophages in the inflamed pancreas of *Rag1*-/- mice, which lack T- and B-cells. The defective macrophage polarization resulted in a severely impaired acinar cell regeneration after induction of pancreatitis (111). Thus, alternatively activated macrophages are not only drivers of fibrosis but also essential for tissue regeneration (110). Which makes treatment more complex! This suggests a critical role of Th2-cells for macrophage polarisation into the alternatively activated phenotype. A general regulator of CD4^+^ T-helper cells are regulatory T-cells. T_regs_ have immunosuppressive activity and are particularly increased in the chronically inflamed pancreas (33–35), where they inhibit Th2-cells and ILC2s. The depletion of Tregs resulted in uncontrolled Th2/ILC2s activation and excessive fibrosis of the pancreas (35). The induction of Tregs could help to prevent excessive fibrosis and one possibility to support the Tregs in their suppressive activity is the administration of IL-2 (123, 124). Interleukin 2, a known T-cell growth factor, can induce and stabilize the Tregs homeostasis (125). However, during the acute phase, excessive activity of Tregs acts also immunosuppressive and can promote the occurrence of infected necrosis (47). Another possibility for a therapeutic intervention with this signaling pathway is the inhibition of IL-4 and IL-13 secretion from their cellular sources. IL-4/IL-13 are primarily secreted by Th2-cells or ILC2s, after their migration into the damaged organ. OC000459 is a CRTH2 antagonist that inhibits migration as well as activation of Th2 cells and ILC2s (126). In clinical studies OC000459 was successfully tested in the treatment of asthma (127). Animal data showed that the inhibition of Th2/ILC2 activation reduced the release of IL-4 and IL-13 in the pancreas and inhibited fibrosis (35). The T-cell/macrophage axis just recently came into the focus of pancreatitis research and is still largely not understood. In addition to Th2 cells, also Th22 and Th17 cells are found in the pancreas (33, 34) and seem to have an impact on fibrosis. Beside CD4^+^ T-helper cells, CD8^+^ T-cells are also present in the pancreas (35), but little is known about their function. Experimental studies on liver fibrosis showed that CD8^+^ cells can act fibrolytic by inducing hepatic stellate cell apoptosis (128) or, like shown for dermal fibrosis, secrete IL-4/IL-13 themselves and further enhance fibrosis (129).

### 3.3 Macrophage independent activation of PSCs

Th22 cells are involved in progressive fibrosis via the secretion of Interleukin 22, which stimulates PSCs to secrete collagen without activating them (130). Likewise, Th17 cells secrete IL-17A which activates PSCs directly via the IL17 receptor and induces the synthesis and secretion of collagen (131). Both cytokines can be depleted by antibodies, which are already approved for the treatment of psoriasis: Fezakinumab, a monoclonal IL-22 antibody (132) and Secukinumab an antibody raised against IL-17A (133). However, IL-22 and IL-17A have also a crucial role in maintaining the intestinal barrier and regulate the secretion of anti-microbial peptides. IL-22 has been demonstrated to be protective in acute pancreatitis, therefore a long-term blockade of these signaling pathways in patients with chronic pancreatitis could have opposing effects, especially in case of an acute pancreatitis episode (134). The currently available data suggest that the treatment of fibrosis in chronic pancreatitis is complex and must consider the involvement of multiple cells of different origin. A general signaling pathway that can be easily manipulated has not yet been identified.

The preventive treatment of fibrosis currently seems not possible without simultaneously affecting cell regeneration as it is the very same growth factors that drive fibrosis as well as regeneration. In addition, also pro-inflammatory mediators such as TNFα and IL-1β can activate pancreatic stellate cells (112, 113), suggesting that fibrosis is initiated already during the acute phase of pancreatitis. The wound healing process is started during pro-inflammation, directly after the onset of pancreatitis. Consequently there exist many different mechanisms that drive fibrosis, like pro-inflammatory mediators IL-1β, IL-6, IL17A, and TNF-α, which can activate stellate cells in addition to the other classical growth factors like TGF-β, FGF, and PDGF (35, 114). This makes an anti-fibrotic therapy a challenging task which should be carefully considered. Ultimately, the most effective treatment of fibrosis should be aimed at minimizing the initial pancreatic damage as early as possible to counteract the development of a necrosis-fibrosis sequence at the outset.

## 4 Conclusion

The most promising therapeutic options for patients suffering from acute and chronic pancreatitis are currently targeted against the involved immune response mechanisms. The inflammatory response is on the other side also a cause of the clinical picture, so a therapeutic approach may address both symptomatic and causal aspects. Previous therapeutic studies which used to target the known triggering pathway of intracellular trypsinogen activation were unsuccessful because these processes are, at the time point of admission to the clinic, normally beyond a point were an intervention could still prevent acinar cell death and the induction of an inflammatory response. Only in a small proportion of patients with confirmed genetic diagnosis a targeted prophylactic therapy which improves protein misfolding or supports intra-acinar protease/anti-protease homeostasis could prevent severe acute episodes and delay disease progression (135).

In all different etiologies of AP it is likewise necessary to prevent or mitigate a severe course of the disease! A strong inflammatory response significantly influences the disease severity and is also responsible for complications like bacterial infected necrosis during the disease course. Targeting a derailing immune response currently appears as the most promising treatment option. Since both pro- and anti-inflammatory mechanisms (SIRS/CARS) can have detrimental influences on disease severity, beneficial therapies must successfully rebalance this system which requires rapid and controllable working tools.

In a significant proportion of AP-patients we see a progress from recurrent episodes to CP, which underlines the need for early therapeutic intervention. While anti-inflammation is shown to be partially protective in the course of acute pancreatitis, it`s pro-fibrotic effects must be considered in recurrent and chronic forms of the disease, as acute and chronic pancreatitis have common underlying disease mechanisms. More experimental studies are needed to unravel the complex immunomodulatory mechanisms involved in the manifestation of the disease in order to uncover further therapeutic options. In parallel, clinical trials should further evaluate the use of already established immune modulating therapeutics.

## Author contributions

MS: Conceptualization, Funding acquisition, Writing – original draft. JG: Conceptualization, Funding acquisition, Writing – original draft, Writing – review & editing. SA: Conceptualization, Writing – original draft. RL: Conceptualization, Writing – original draft. HM: Conceptualization, Writing – original draft. HE: Conceptualization, Writing – original draft. FW: Conceptualization, Writing – original draft, Writing – review & editing.
